# Society Notes

**Published:** 1901-12

**Authors:** 


Secretary.

---

### SOCIETY NOTES.

Dr. John J. Repp, of Ames, Iowa, succeeds Dr. John E. Brown, of Oskaloosa, the same State, as Secretary of the Iowa State Veterinary Medical Association.

Secretary Ranck, of the Keystone Veterinary Medical Association, should be very proud of the interesting meeting in December of that Assotion. The addresses of Drs. Pearson and Ravenel were admirable and the attendance unusually large.

The annual meeting of the Minnesota State Veterinary Medical Association will be held in St. Paul on January 15 and 16, 1902. The clinical work will be under the direction of Dr. M. H. Reynolds at the State Experimental Station.

